# Translational Medicine in Pulmonary-Renal Crosstalk: Therapeutic Targeting of p-Cresyl Sulfate Triggered Nonspecific ROS and Chemoattractants in Dyspneic Patients with Uremic Lung Injury

**DOI:** 10.3390/jcm7090266

**Published:** 2018-09-09

**Authors:** Jia-Feng Chang, Shih-Shin Liang, Pounraj Thanasekaran, Hsueh-Wei Chang, Li-Li Wen, Chung-Hua Chen, Jian-Chiun Liou, Jih-Chen Yeh, Shih-Hao Liu, Huei-Min Dai, Wei-Ning Lin

**Affiliations:** 1 Program in Nutrition and Food Sciences, College of Human Ecology, Fu Jen Catholic University, New Taipei City 242, Taiwan; 01508@km.eck.org.tw; 2 Graduate Institution of Biomedical and Pharmaceutical Science, College of Medicine, Fu Jen Catholic University, New Taipei City 242, Taiwan; jillkaom@yahoo.com.tw; 3 Division of Nephrology, Department of Internal Medicine, En-Chu-Kong Hospital, New Taipei City 237, Taiwan; 4 Renal Care Joint Foundation, New Taipei City 220, Taiwan; 5 Division of Nephrology, Department of Internal Medicine, Far Eastern Memorial Hospital, New Taipei City 220, Taiwan; 6 Department of Nursing, Yuanpei University of Medical Technology, Hsinchu 300, Taiwan; 7 Institute of Biomedical Science, National Sun Yat-Sen University, Kaohsiung 804, Taiwan; liang0615@kmu.edu.tw; 8 Department of Biotechnology, College of Life Science; Center for Research, Resources and Development, Kaohsiung Medical University, Kaohsiung 807, Taiwan; 9 Institute of Chemistry, Academia Sinica, Taipei 115, Taiwan; ptsekaran@gmail.com; 10 Department of Biomedical Science and Environmental Biology, Kaohsiung Medical University, Kaohsiung 807, Taiwan; changhw@kmu.edu.tw; 11 Department of Medical Laboratory Science and Biotechnology, Yuanpei University, Hsinchu 300, Taiwan; eckwen@yahoo.com; 12 Department of Clinical Laboratory, En Chu Kong Hospital, New Taipei City 237, Taiwan; 13 Department of Chest Medicine, En Chu Kong Hospital, New Taipei City 237, Taiwan; 01273@km.eck.org.tw; 14 School of Biomedical Engineering, Taipei Medical University, Taipei 110, Taiwan; jcliou@tmu.edu.tw; 15 Department of Dentistry, Far Eastern Memorial Hospital, New Taipei City 220, Taiwan; b202093012@tmu.edu.tw; 16 Division of Pathology, En-Chu-Kong Hospital, New Taipei City 237, Taiwan; 01393@km.eck.org.tw

**Keywords:** *p*-cresyl sulfate, alveolar cell death, reactive oxygen species, cytokines, uremic lung injury, chronic kidney disease

## Abstract

Molecular mechanisms and pathological features of p-Cresyl sulfate (PCS)-induced uremic lung injury (ULI) in chronic kidney disease (CKD) remain unclear. We analyzed pleural effusions (PE) from CKD and non-CKD patients for uremic toxins, reactive oxygen species (ROS), and chemotactic cytokines. Correlations between PE biomarkers and serum creatinine were also studied. Cell viability and inflammatory signaling pathways were investigated in PCS-treated human alveolar cell model. To mimic human diseases, CKD-ULI mouse model was developed with quantitative comparison of immunostaining and morphometric approach. PE from CKD patients enhance expressions of uremic toxins, hydroxyl radicals, and IL-5/IL-6/IL-8/IL-10/IL-13/ENA-78/GRO α/MDC/thrombopoietin/VEGF. PE concentrations of ENA-78/VEGF/IL-8/MDC/PCS/indoxyl sulphate correlate with serum creatinine concentrations. In vitro, PCS promotes alveolar cell death, cPLA2/COX-2/aquaporin-4 expression, and NADPH oxidase/mitochondria activation-related ROS. Intracellular ROS is abrogated by non-specific ROS scavenger N-acetyl cysteine (NAC), inhibitors of NADPH oxidase and mitochondria-targeted superoxide scavenger. However, only NAC protects against PCS-induced cell death. In vivo, expressions of cPLA2/COX2/8-OHdG, resident alveolar macrophages, recruited leukocytes, alveolar space, interstitial edema and capillary leakage increase in lung tissues of CKD-ULI mice, and NAC pretreatment ameliorates alveolar–capillary injury. PCS causes alveolar–capillary injury through triggering intracellular ROS, downstream prostaglandin pathways, cell death, and activating leukocytes to release multiplex chemoattractants and extracellular ROS. Thus PCS and nonspecific ROS serve as potential therapeutic targets.

## 1. Introduction

Respiratory distress due to pulmonary edema is a life-threatening complication in patients with renal failure [1]. Cardiogenic pulmonary edema resulting from elevated capillary hydrostatic pressure can be relieved by fluid removal via ultrafiltration or diuresis immediately [2]. Non-cardiogenic pulmonary exudate is caused by inflammation-related hyperpermeability, yet fluid removal only partially improves oxygenation [3]. Patients with chronic kidney disease (CKD) may fall in the spectrum between cardiogenic and non-cardiogenic pulmonary edema [3]. Emerging evidences demonstrate uremic retention solutes exhibit pro-inflammation and pro-oxidant effects on different cell and organ systems [4]. Myriads of mechanisms are involved in uremic lung injury (ULI), including neutrophil activation, vascular hyperpermeability, dysregulation of salt-water transporters, and cytokine expressions [5,6,7,8,9]. Nonetheless, the majority of experimental designs are animal models of acute kidney injury instead of CKD. Our study elucidates prostaglandin (PG) pathways account for inflammatory lung diseases and leukocyte recruitment [10]. Moreover, aquaporins regulate cell volume and membrane water permeability in alveolar fluid homeostasis [11]. Despite previous documented implications, the potential toxic effects of uremic solutes on ULI remain unclear. 

*p*-Cresyl sulfate (PCS), a protein-bound non-dialyzable uremic toxin, accumulates in CKD patients. PCS derives from secondary metabolism of *p*-cresol, which is produced from protein fermentation by intestinal bacteria [12]. PCS exerts deleterious effects on diverse cell systems: cardiac dysfunction and cardiomyocyte apoptosis resulting from reactive oxygen species (ROS), renal tubular cell damage via NADPH oxidase, ROS production by leukocytes, and endothelial/mononuclear cell cycle arrest due to ROS generation [13,14,15,16]. Clinical researches demonstrate serum concentrations of PCS serve as a predictor of cardiovascular and all-cause mortality in patients with hemodialysis [17,18]. In light of this, PCS might adversely affect the respiratory and immune system. To test this hypothesis, analyzes of uremic toxins, hydroxyl radicals, chemotactic cytokines, and recruited leukocytes in pleural effusions were compared between CKD patients and non-CKD subjects. From bedside to bench, cell viability and inflammatory signaling pathways with reactive oxygen species (ROS) were investigated in PCS-treated human alveolar cell model. To mimic human diseases, we developed a CKD-ULI mouse model using quantitative comparison of immunohistochemical staining and morphometric approach. 

## 2. Materials and Methods

### 2.1. Assays of PCS and Indoxyl Sulfate (IS) in Human Pleural Effusions

The study had been reviewed and approved by the Research Ethics Review Committee of the En Chu Kong Hospital for all bio-clinical specimens (ECKH-IRB-1050102). Human pleural effusions (50 μL) were pretreated by 1400 μL acetonitrile to precipitate proteins. After centrifugation, each supernatant of samples was collected in tube and evaporated by spin vacuum instrument. PCS and IS in lyophilized samples were analyzed by Mass Spectrometer Analytical System (Thermo Fisher Scientific Inc., Waltham, MA, USA) and UHPLC analytical system (Thermo Fisher Scientific Inc., Waltham, MA, USA). The Xcalibur software (version 2.2, Thermo-Finnigan Inc., San Jose, CA, USA) was used for method setup and data processing.

### 2.2. Measurement of Cytokines and Peroxidation in Pleural Effusions

Concentrations of inflammatory cytokines were measured by RayBio® Human ELISA Kit (RayBiotech, Inc., Norcross, GA, USA) of ELH-IL8-1, ELH-MDC-1, ELH-VEGF-1, and ELH-ENA78-1. Peroxidation of pleural effusion was measured indirectly through thiobarbituric acid reaction. The 100 μL pleural effusion was blended with 700 μL of 0.2 M H3PO4, 100 μL of 1 mM FeCl_3_ with 1.04 mM EDTA and 100 μL 10 mM ascorbic acid. And then at 37 °C hot-bath for 10 min, the solution was mixed with 500 μL (1%) thiobarbituric acid and 1000 μL 2.8% TCA reagent. With 8 min 100 °C hot bath, 20 min ice bath and waiting for 10 min at room temperature, the solution was detected by UV spectrometry at 532 nm. The percentage of sample was calculated by a formula as follows: (Absorbance 532 nm sample/Absorbance 532 nm control) × 100%.

### 2.3. Statistical Analysis of Data

All data are expressed as the mean ± SEM of the mean using the GraphPad Prism Program (GraphPad, San Diego, CA, USA) or SPSS (Statistical Package for the Sociological Sciences; IBM, Armonk, NY, USA), version 22.0. Quantitative data were analyzed with one-way analyses of variance (ANOVA) followed by Tukey’s post hoc test or the independent samples *t* test. The significance threshold was set at 5% (*p* < 0.05). All of the experiments were performed at least five times.

## 3. Results

### 3.1. Pleural Effusion Concentrations of Chemotactic Cytokines and Uremic Toxins Correlate with Renal Function Tests; Pleural Effusions from CKD Patients Exert Higher Expressions of Uremic Toxins and Hydroxyl Radicals

**Figure 1:**
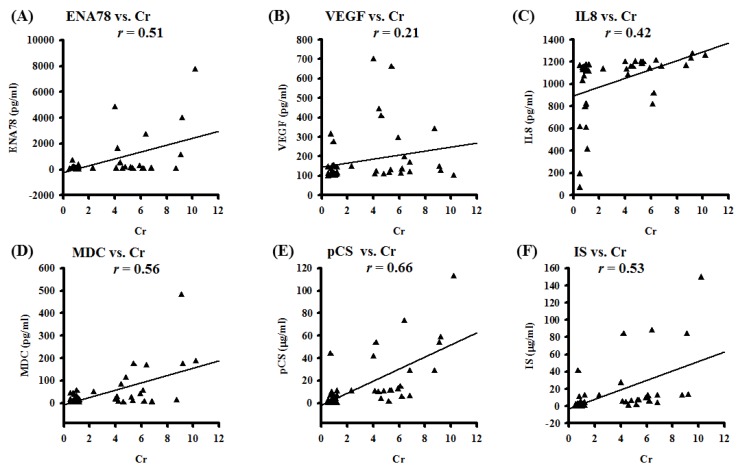
Pleural effusion concentrations of uremic toxins and selected chemotactic cytokines correlate with renal function tests in patients with respiratory distress. (**A**) The correlation coefficient *r* between ENA-78 and serum Cr is 0.51; (**B**) The correlation coefficient *r* between VEGF and serum Cr is 0.21; (**C**) The correlation coefficient *r* between IL8 and serum Cr is 0.42; (**D**) The correlation coefficient *r* between MDC and serum Cr is 0.56; (**E**) The correlation coefficient *r* between PCS and serum Cr is 0.66; (**F**) The correlation coefficient *r* between IS and serum Cr is 0.53.

PCS exerts pro-inflammatory and pro-oxidant effects on multiple organ systems in CKD patients. We aimed to prove pleural effusions from CKD patients may enhance expressions of uremic toxins, extracellular ROS, and chemotactic cytokines, leading to alveolo-capillary injury. Flow chart of study patient enrollment was shown in Supplementary Figure S1. The background bio-demographic characteristics were similar except the renal function related profiles between selected uremic and non-uremic patients. As expected, pleural effusions from CKD patients exhibited higher concentrations of PCS and indoxyl sulphate (IS), associated with an increment of proteinaceous fluid leakage and hydroxyl radicals (Supplementary Table S1 and Figure S2A). To screen which types of cytokines were involved in the mechanism of CKD-ULI, the human cytokine array was used to detect 42 types of inflammatory cytokines. Compared with the control group of non-CKD patients with pure cardiogenic pulmonary edema, uremic pleural effusions obtained from dyspneic patients with CKD exerted higher expression of various cytokines, including IL-5, IL-6, IL-8, IL-10, IL-13, epithelial-derived neutrophil-activating peptide 78 (ENA-78), macrophage-derived chemokine (MDC), thrombopoietin, vascular endothelial growth factor (VEGF), and growth-related oncogene-α (GRO-α) (Supplementary Figure S2B). To investigate whether uremic toxins and chemotactic cytokines from pleural effusions were associated with renal function, we conducted a correlation analysis between selected pleural biomarkers and serum creatinine (Cr). The scatter diagram indicated correlations between ENA-78/VEGF/IL-8/MDC/PCS/IS and Cr were robust (Figure 1). Positive correlations between pleural uremic toxins and serum Cr strongly suggest that exchanges between both compartments can occur in both directions. In light of this, pleural levels of uremic toxins and cytokines may reflect systemic inflammation due to uremic burden. Above results unveiled uremic toxins-related extracellular ROS, chemoattractants, and systemic immune responses may mediate pulmonary-renal crosstalk, but underlying mechanisms and inflammatory signaling pathways remain unelucidated.

Pleural effusion concentrations of PCS and IS were evaluated by Mass Spectrometer (Thermo Finnigan TSQ Quantum Ultra Mass Spectrometer, Thermo Fisher Scientific Inc., Waltham, MA, USA). Pleural effusion concentrations of selected inflammatory cytokines were detected by RayBio® Human ELISA Kit of ELH-IL8-1, ELH-MDC-1, ELH-VEGF-1, and ELH-ENA78-1. Data of the correlation coefficients are represented by *r*; *n* = 42.

### 3.2. PCS Promotes Alveolar Cell Death in a Time- and Dose-Dependent Manner

**Figure 2:**
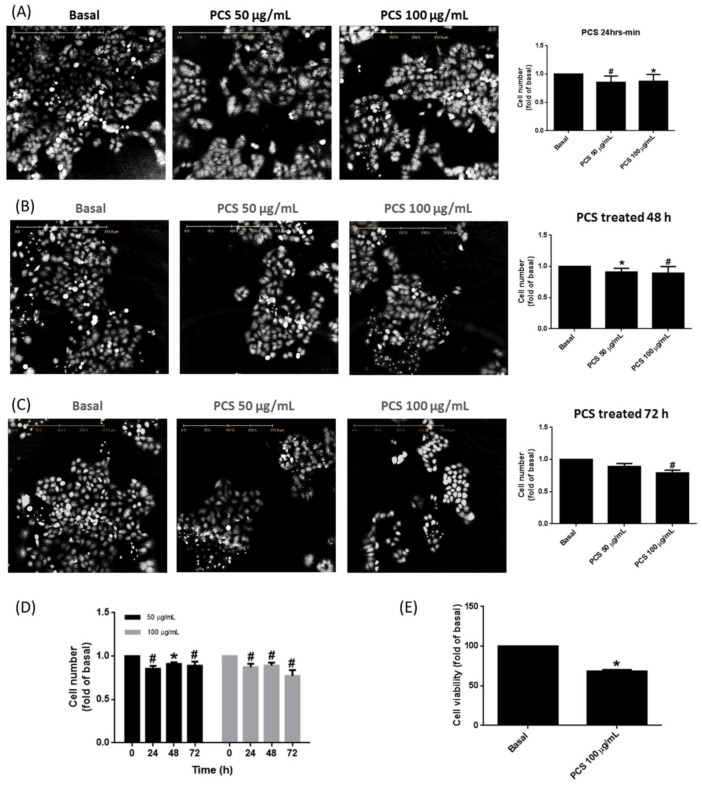
p-Cresyl sulfate (PCS) promotes human pulmonary alveolar cell death in a time- and dose-dependent manner. Serum-starved A549 cells were treated without or with PCS (50 or 100 μg/mL) for (**A**) 24 h, (**B**) 48 h, and (**C**) 72 h. (**D**) cells were treated with 50 or 100 μg/mL PCS for 0, 24, 48, or 72 h. At the end of incubation, holographic images were captured at least from five random areas. Cell number changes were analyzed by HoloStudio software. (**E**) Cell viability was analyzed by XTT assay according to the direction of manufacturer. Absorbance was measured at 490 nm and 650 nm using a BioTek spectrophotometer. Data are expressed as mean ± SEM of different independent experiments (*n* > 5). * *p* < 0.05, ^#^ *p* < 0.01 as compared with the groups of Basal or 0 min.

To outreach above findings to basic research, cell viability was evaluated by holographic imaging cytometry (HoloMonitor M4) or tetrazolium salt (XTT) assay in PCS-treated human alveolar cell model over a 72-h window. As shown in Figure 2A, quantitative analysis elucidated PCS suppressed A549 cell viability in a time- and concentration-dependent manner. Figure 2B,C illustrated A549 cell death significantly reached maximum at 72 h. As shown in Figure 2D with 3D histogram, A549 cells exposed to 100 μg/mL PCS for 72 h exerted the highest death rate.

### 3.3. Non-Specific Intracellular ROS Involves in PCS-Induced Alveolar Cell Death

**Figure 3:**
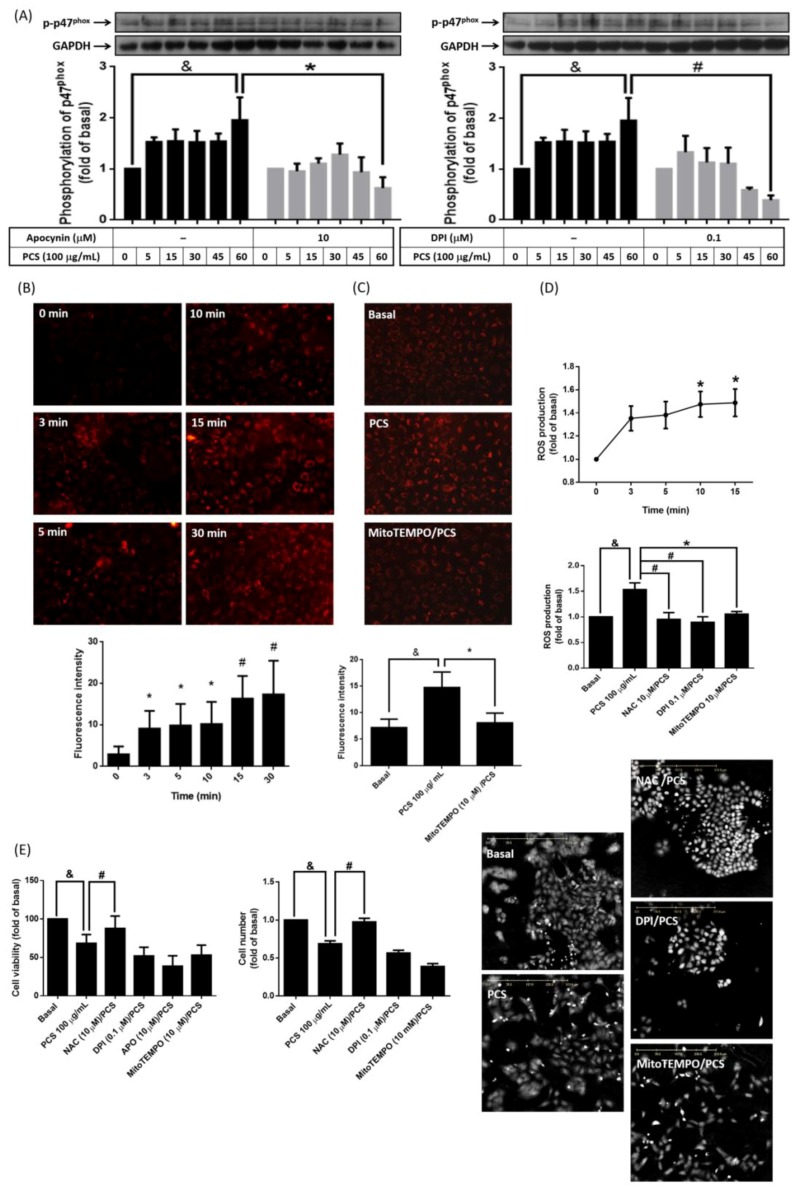
Intracellular reactive oxygen species (ROS) involve in PCS-induced human pulmonary alveolar cell death. (**A**) Cells were treated without or with apocynin (APO, 10 μM) or diphenylene iodonium (DPI, 0.1 μM) for 1 h, and then incubated with 100 μg/mL PCS for the indicated time points. Phosphorylation of p47^phox^ protein (NADPH oxidase subunit) was determined by Western blot; (**B**) Cells were treated with 100 μg/mL PCS for indicated time intervals and labeled by MitoSOX^TM^ Red. The fluorescence images of cells were captured by a fluorescence microscope; (**C**) Cells were pretreated with MitoTEMPO (10 μM) for 1 h, and then incubated with PCS for another 30 min. Images were acquired by a fluorescence microscope after MitoSOX^TM^ Red labeling; (**D**) Cells were treated with 100 μg/mL PCS for indicated time intervals. Or cells were pretreated without or with N-acetyl-L-cysteine (NAC, 10 μM), APO (10 μM), or DPI (0.1 μM) for 1 h and then incubated with 100 μg/mL of PCS for another 15 min. Intracellular ROS was determined by H2DCFDA staining assay; (**E**) Cells were pretreated without or with NAC (10 μM), APO (10 μM), DPI (0.1 μM) or MitoTEMPO (10 μM) for 1 h, and then incubated with 100 μg/mL PCS for 72 h. Cell viability and cell number were analyzed by XTT assay and Holographic cell analysis. Data are expressed as line chart or mean ± SEM of different independent experiments (*n* > 4); ^&^ *p* < 0.05; * *p* < 0.05; and ^#^ *p* < 0.01 to compare the differences between the two indicated groups or compare with the group of 0 min.

Cytotoxic effects of PCS are attributed to up-regulated NADPH oxidase activity [13,14], and mitochondrial dysfunction [19,20]. To investigate the mechanism further, phosphorylation of p47^phox^ protein was determined with or without the treatment of NADPH oxidase inhibitor (APO (10 μM) or DPI (0.1 μM)). We found PCS induced phosphorylation of p47^phox^ in a time dependent manner (Figure 3A). Pretreatment of APO and DPI attenuated p47^phox^ phosphorylation in PCS-stimulated cells (Figure 3A). On the aspect of mitochondria activation, A549 cells stained with MitoSOX^TM^ Red were continuously exposed to 100 μg/mL PCS for 30 min. Figure 3B showed a sustained rise in mitochondrial ROS generation was detected from 3 min to 30 min. Such PCS-induced mitochondrial ROS increment was inhibited by pretreatment of mitochondrial superoxide scavenger (MitoTEMPO) (Figure 3C). Further, PCS-induced intracellular ROS reached the peak within 15 min, which was attenuated by pretreatment of non-specific ROS scavenger (NAC) (Figure 3D). Similarly, pretreatment of NADPH oxidase (DPI), or mitochondrial superoxide scavenger (MitoTEMPO) significantly reduced PCS-induced ROS accumulation (Figure 3D). To investigate whether intracellular ROS involved in PCS-modulated alveolar cell death, A549 cells were pretreated with or without APO (10 μM), DPI (0.1 μM), MitoTEMPO (10 μM) or NAC (10 μM) for 1 h, and then incubated with 100 μg/mL PCS for 72 h. APO, DPI together with MitoTEMPO did not reverse pro-death effect of PCS in a 72 h time window (Figure 3E), but pretreatment of NAC significantly reversed PCS-induced cell death. Current data demonstrate that intracellular sources of ROS contributed to PCS-promoted alveolar cell death were nonspecific and across-the-board, instead of single origin from NADPH oxidase or mitochondria.

### 3.4. PCS Enhances Expression of cPLA2/COX-2 and Aquaporin-4 in Alveolar Cells

We have reported NADPH oxidase- and mitochondria-derived ROS activate the expression of downstream genes of cPLA2 and COX-2 mediating leukocyte recruitment responding to pulmonary inflammation [10], and aquaporins participated in apoptosis [21,22]. However, whether PCS modulated expressions of cPLA2, COX-2, and AQP4 remain unclear. As shown in Supplementary Figure S3A, PCS induced protein expressions of cPLA2 and COX-2 were significantly increased with higher concentrations of PCS (100 μg/mL) within 24 h and reached maximum at 6 h. As shown in Supplementary Figure S3B, reverse transcription polymerase chain reaction (RT-PCR) demonstrated that PCS induced mRNA accumulation of cPLA2 and COX-2 in a time-dependent manner. To investigate whether PCS enhanced alveolar cell hyperpermeability during pulmonary edema in human disease of CKD-ULI, we additionally investigated the effect of PCS on the expression of aquaporin-4 in our cell model. Supplementary Figure S3C unveiled PCS accentuated AOP4 expression in a time- and concentration-dependent manner. AOP4 expression was significantly increased with higher concentrations of PCS (100 μg/mL) and reached maximum at 24 h. This finding suggested that PCS-induced cPLA2, COX-2, and AOP4 expression enhanced alveolar cell injury during CKD-ULI. While considering nephrotoxic effects of COX-2 inhibitors for CKD patients in clinical practice, we investigated the E series of PG receptors as downstream effectors of COX-2 and cPLA2 pathways. Studies indicate PGE2 mediates cell migration and COX-2–dependent ROS signaling pathways in pathophysiology of respiratory diseases [23,24], but effects of PGE2 on PCS-modulated alveolar cell viability remain unclear. A549 cells were pretreated with or without 10 μM of sc-51089 (inhibitor of PGE2 receptor), and then incubated with 100 μg/mL PCS for 72 h. As shown in Supplementary Figure S3D, 100 μg/mL PCS decreased A549 cell viability while 10 μM of sc-51089 did not reverse pro-death effects of PCS. Thus pro-death effects of PCS on A549 cell were not limited to PG pathways.

### 3.5. PCS Increases Alveolar Space and Enhances Cell Death with Expressions of COX-2, cPLA2 and ROS in Lung Tissues of CKD-ULI Mouse

**Figure 4:**
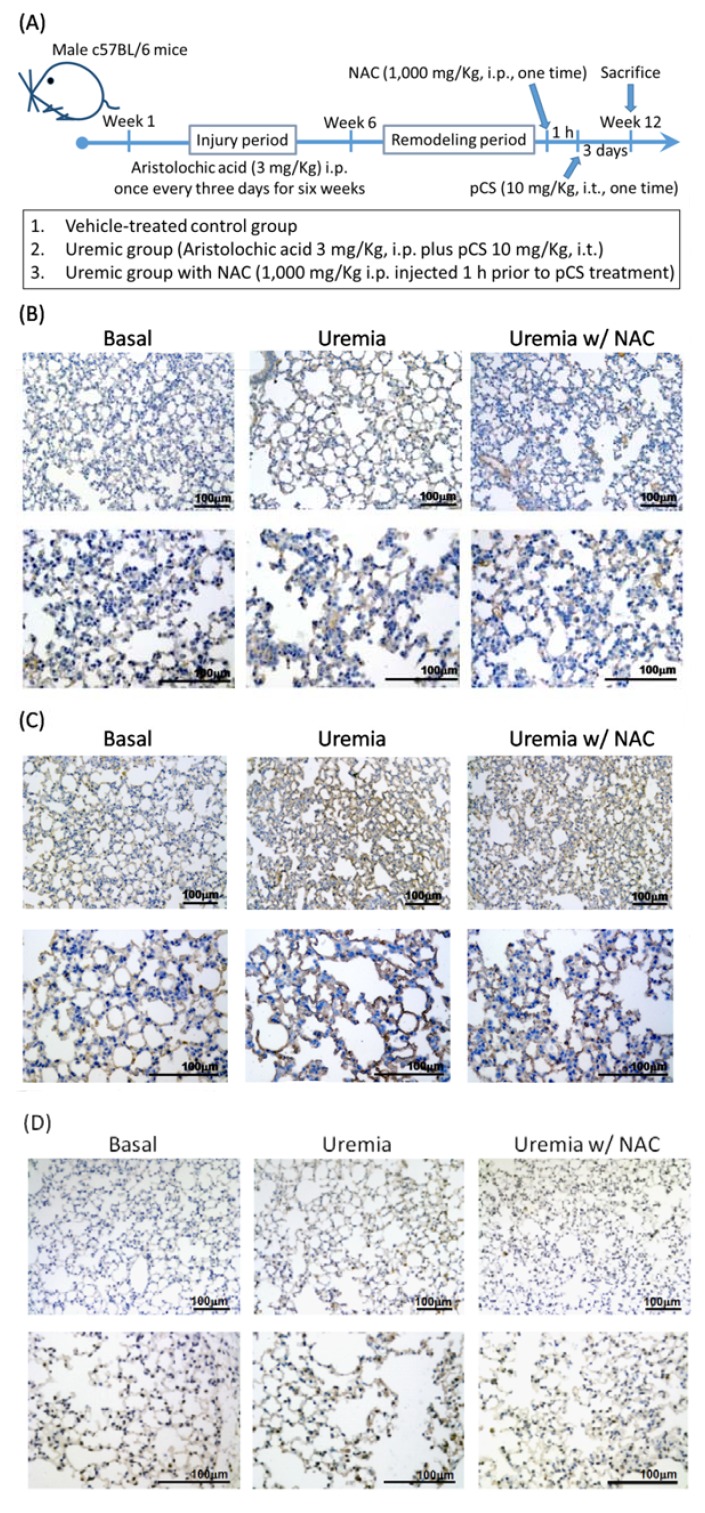

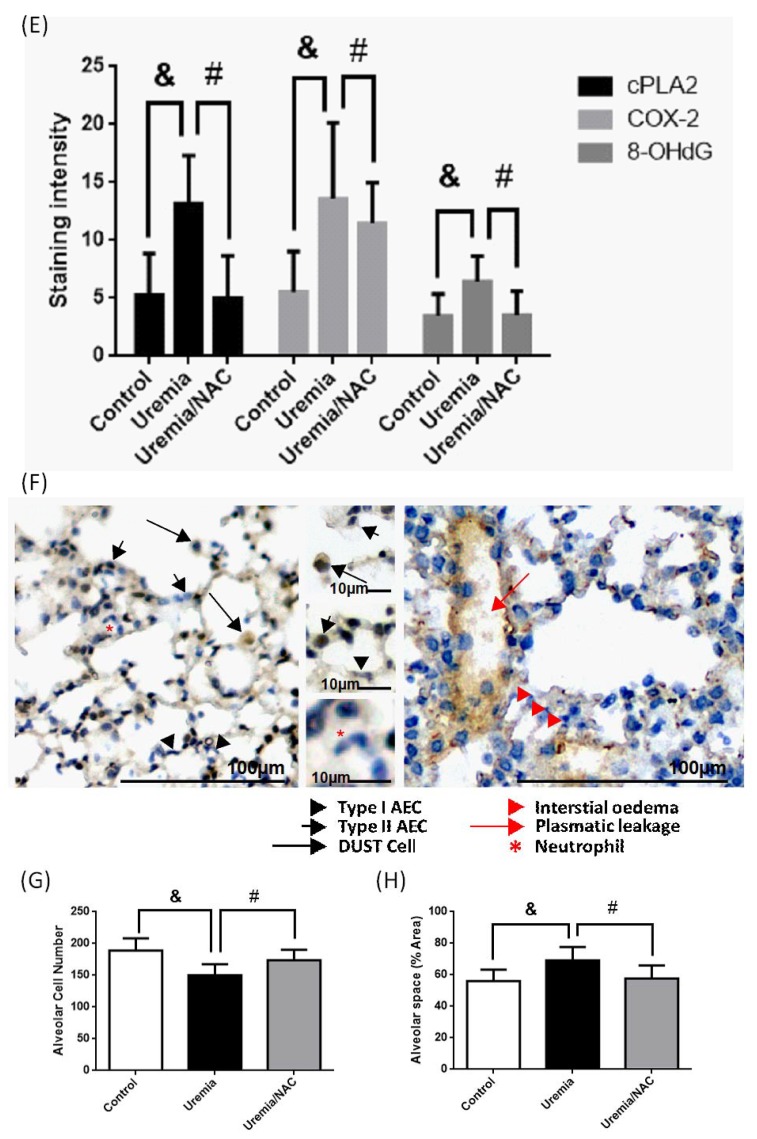
PCS enhances the expressions of COX-2/cPLA2 and ROS and alveolar cell death in uremic lung tissues of CKD mice. (**A**) Working model of CKD-ULI. Mice were randomized into three groups: vehicle-treated control group (normal renal function), uremic group, and uremic group with inhibitor (NAC) treatment. The uremic group was induced with aristolochic acid nephropathy and PCS (10 mg/kg i.t., one time for 3 days before sacrificed) was placed posterior in the throat with the support of otoscope and aspirated into lungs at week 12. For uremia/NAC group, NAC (1000 mg/kg) was i.p. injected 1 h prior to PCS treatment. At the end of treatment, mice were scarified and lung tissues were extracted and paraffinized. immunohistochemical stain was performed to detect (**B**) cPLA2, (**C**) COX-2, and (**D**) 8-OHdG. (**E**) Quantification of immunohistochemical images were performed by image J. (**F**) Closer images of immunohistochemical stain to show the indicated cells or phenomena. Quantification of alveolar cell number (**G**) and alveolar space (**H**). Data are expressed as mean ± SEM (*n* = 5); ^&^ *p* < 0.05 and *^#^ p* < 0.01 to compare the differences between the two indicated groups.

Since we proved PCS enhanced expressions of COX-2/cPLA2 and ROS in vitro, we aimed to investigate above effects in vivo. The setup of CKD-ULI mice model was modified from previous described and shown in Figure 4A [25]. At week 12, mice were sacrificed and immunohistochemical stain was used to analyze protein expressions of cPLA2, COX-2, or 8-OHdG of lung tissue. As shown in Figure 4B–E, lung tissues from CKD-ULI mice (the uremia groups) possessed higher protein expressions of cPLA2, COX-2, and 8-OHdG at the end of treatment. Non-specific antioxidant treatment attenuated such protein expressions in lung tissues from the uremia/NAC groups. In a morphometric approach, all the uremia groups presented diffusely dilated alveolar space (reduced alveolar cell number), more alveolar macrophages (dust cells), neutrophils, interstitial edema, and plasmatic leakage into the alveoli and alveolar ducts (Figure 4F). Figure 4G and 4H illustrated the comparison of quantified analysis for alveolar cell number and alveolar space. The above findings indicate the fact that the pro-inflammatory and pro-oxidant effects of PCS cause alveolar-capillary injury, and immune system may participate in the pulmonary-renal crosstalk of CKD-ULI. 

## 4. Discussion

Mechanisms of CKD-ULI mediated by diverse classes of signaling pathways and responding cells are so intricate that effective therapies are still lacking. From the view of translational medicine in pulmonary-renal crosstalk, we integrate human samples, cell and mouse models to explore how uremic toxins impair alveolar-capillary integrity. Several important issues in this research deserve further discussion.

Our in vitro data demonstrated non-specific intracellular ROS participated in PCS-induced alveolar cell death. Although intracellular ROS was abrogated by inhibitors of NADPH oxidase and mitochondria-targeted superoxide scavenger, single and specific inhibition of mitochondrial or NADPH oxidase pathway could not reverse pro-death effects of PCS. Thus intracellular sources of ROS induced by PCS are multifaceted and nonspecific, leading to a strong ROS. The only way to reverse the cell death is scavenging the total intracellular ROS by nonspecific antioxidants. Likewise, PCS promotes mRNA and protein expression of cPLA2 and COX-2 in alveolar cells, yet single inhibition of PG pathways is unable to reverse PCS- induced cell death. Thus PCS-induced intracellular inflammatory signaling pathways are diverse and intermixed. Our team has indicated ROS is a key messenger of signaling cascades in pulmonary diseases that responds to various extracellular stimuli via targeting transcription factors, modulating inflammatory gene expression and target proteins, such as COX-2, and cPLA2 [26]. Interestingly, PCS-induced intracellular ROS reach the peak within 15 min, which is far faster than the driving time of mRNA/protein expression of cPLA2 and COX-2. In light of this, multiplex origins of ROS may account for second messenger molecules in response to PCS exposure that triggering downstream inflammatory signaling cascades to accelerate alveolar cell death.

Under inflammatory conditions, ROS dramatically increases and overwhelmed antioxidant systems, resulting in subsequent alteration of membrane lipids, proteins, and nucleic acids [27]. Thus we investigated levels of 8-OHdG and PG pathways in mouse lung tissue to provide the first comprehensive analysis for pathomechanisms of CKD-ULI. As expected, lung tissues from the uremia groups enhanced expressions of cPLA2, COX-2, and 8-OHdG. Surprisingly, NAC attenuated COX2 inhibition only oxidative injury (8-OHdG) but also inflammatory protein expressions (cPLA2/COX-2). The in vivo data support our in vitro data that ROS acts as second messenger molecules in response to PCS exposure that trigger downstream inflammatory signaling cascades (PG pathways). In morphometric analysis, lung tissues from CKD-ULI mice present decreased alveolar cell number, diffusely dilated alveolar space, plasmatic leakage, interstitial edema, and more recruited leukocytes, supporting the evidence of alveolar–capillary injury. Most important of all, this is the first CKD mouse model that expresses a complete picture of ULI, instead of distant organ effects due to acute kidney injury [1]. 

Leukocytes activation plays a pivotal role in generating ROS and augmenting lung inflammation [28,29]. Disruption of vascular endothelial barrier due to inflammatory cytokines and ROS result in proteins, fluid, and immune cells across vessels into tissues during lung inflammation [30]. In our research, stronger extracellular ROS may arise from the superimposition of recruited leukocytes on activated pulmonary macrophages, resulting in worse proteinaceous fluid leakage and alveolo-capillary injury. Our human data illustrate various pro-adhesive factors (VEGF and thrombopoietin) and chemotactic cytokines (IL-5, IL-6, IL-8, IL-10, IL-13, ENA-78, MDC, and GRO-α) are associated with above leukocyte recruitment. We firstly prove pleural effusions from CKD patients with respiratory distress enhance expressions of uremic toxins, multiplex chemoattractants and extracellular ROS. These novel findings partially explain intricate mechanisms in pulmonary-renal crosstalk, providing the first clinical database for future development of cytokine inhibitors. 

## 5. Conclusions

**Figure 5:**
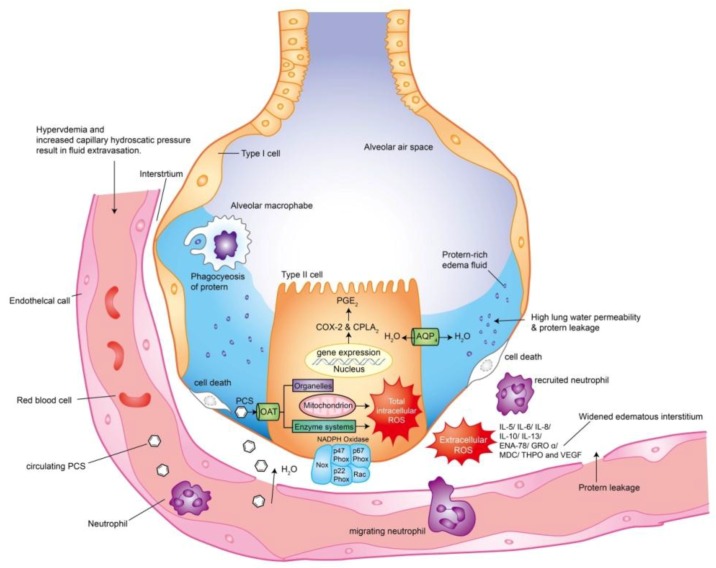
Intricate pathomechanisms of uremic lung injury in chronic kidney disease: Intra-alveolar and extra- alveolar cell responses.

Our research has contributed a mechanistic insight of CKD-ULI, showing that PCS impairs alveolar–capillary integrity through triggering intracellular ROS, activating downstream PG pathways, cell death, and recruiting leukocytes to release extracellular ROS and multiplex chemoattractants (Figure 5). PCS may serve as a predisposition factor of ULI and thus a therapeutic target. We also elucidate non-specific antioxidant NAC prevents alveolar cell death and attenuates lung tissue damages in the uremic milieu. This potential antidote can easily be applied to clinical practice, because oral large dose of NAC is well tolerated without systemic side effects. In light of the growing prevalence of CKD worldwide with an increasing trend in total medicare expenditures, the organ-protective effects of NAC should be tested in uremic patients with urgent need of new therapeutics. Future therapeutic strategy for CKD-ULI should focus on combined PCS-lowering agents and strong nonspecific antioxidants.

The schematic diagram has contributed a mechanistic insight of pulmonary-renal crosstalk in chronic kidney disease (CKD) patients with respiratory distress. PCS induces uremic lung injury (ULI) through triggering intracellular ROS, activating downstream PG pathways, cell death, and recruiting leukocytes to release ROS and multiplex chemoattractants. For intra-alveolar cell response, ROS may account for second messenger molecules after PCS exposure that triggering downstream inflammatory signaling cascades to accelerate alveolar cell death. For extra-alveolar cell responses, ROS and inflammatory cytokines contribute to alveolar–capillary injury and proteinaceous fluid leakage. PCS and nonspecific antioxidants may serve as predisposition factors and thus therapeutic targets in preventing CKD-ULI.

## Supplementary Materials

The following are available online at http://www.mdpi.com/2077-0383/7/9/266/s1, Figure S1: The flow chart of patient enrollment, Figure S2: (**A**) Pleural effusions from uremic patients exert higher concentrations of p-Cresyl sulfate (PCS), indoxyl sulphate (IS), and hydroxyl radicals than those from patients with pure cardiogenic pulmonary edema; (**B**) Enhanced expressions of multiplex human inflammatory cytokines in uremic pleural effusions. Relative expressions of human inflammatory cytokines were detected by RayBio® G-Series Human Cytokine Antibody Array 3 Kit, Figure S3: PCS enhances protein and mRNA expression of cPLA2, COX-2 and aquaporin-4 in human pulmonary alveolar cells, Table S1: Comparisons of background bio-demographic characteristics and relevant laboratory data between selected uremic and non-uremic patients with pleural effusions.
