# Supplementary material for: Translational Medicine in Pulmonary-Renal Crosstalk: Therapeutic Targeting of p-Cresyl Sulfate Triggered Nonspecific ROS and Chemoattractants in Dyspneic Patients with Uremic Lung Injury
Source: J Clin Med. 2018 Sep 9;7(9):266. doi: 10.3390/jcm7090266 (PMC6162871; doi:10.3390/jcm7090266)
Supplement: Supplementary file 1 [file jcm-07-00266-s001.pdf]

Online Data Supplement

Materials

Most materials and methods were previously described [1]. Other materials can be purchased from Santa Cruz Biotechnology (Santa Cruz, CA, USA) or Sigma (St. Louis, MO, USA), including inhibitor of PGE2 receptor (sc-51089), N-acetylcysteine, apocynin (APO), diphenylene iodonium (DPI) and MitoTEMPO. Cytokine antibody array was from Ray BioHuman (RayBiotech, Norcross, GA). C57BL/6 mice were from the National Laboratory Animal Center (Taipei, Taiwan).

Cell culture and treatments

A549 cells, human type II alveolar epithelial cell, were cultured as previously described [1]. PCS (Kureha, Tokyo, Japan) was dissolved in 0.1% BSA. All inhibitors were dissolved in DMSO except that NAC was in distilled water. Serum-starved cells were incubated with PCS for the various concentrations and time intervals. If inhibitors or antibody were used, they were added 1 h before the treatment of PCS, including sc-51089 (inhibitor of PGE2 receptor, 10 μM), NAC (ROS scavenger, 10 μM), DPI (inhibitor of NADPH oxidase, 0.1 μM), Apo (inhibitor of NADPH oxidase, 10 μM) or MitoTEMP (mitochondria-targeted superoxide scavenger, 10 μM).

Animal treatment

Six-week-old male C57BL/6 mice were studied according to the guidelines of the Animal Care Committee of Fu Jen Catholic University and National Institute of Health Guides for the Care and Use of Laboratory Animals. Mice were randomized into three groups: vehicle-treated control group (normal renal function), uremic group, and uremic group with radical scavenger NAC treatment (Uremia/NAC group). We developed a standardized protocol of CKD-ULI mouse model to induce tubulointerstitial fibrosis using intraperitoneal (i.p.) injection of pure aristolochic acid I (AAI, 3 mg/kg i.p., once every three days for six weeks followed by a CKD development time of 6 weeks) and intratracheal (i.t.) administration of PCS (10 mg/kg i.t., one time for 3 days before sacrificed) [2-4]. The protocol of i.t. administration was described previously [1]. PCS was placed posterior in the throat with the support of otoscope (Heine Optotechnik, Dover, DE) and aspirated into lungs at week 12. For uremia/NAC group, NAC (1,000 mg/Kg) was i.p. injected 1 h prior to PCS treatment. At the end of treatments, lung tissues were removed for protein analysis with immunohistochemical stain.

Protein extraction and Western blot

After PCS stimulation, the cells were then rapidly washed with ice-cold PBS, scraped, and collected by centrifugation at 1000 × g for 10 min. The collected cells were lysed with ice-cold lysis buffer. The lysates were centrifuged at 4500 × g for 1 h at 4 °C to yield the whole cell extract. Samples from these supernatant fractions (30 μg protein) were subjected to SDS-PAGE using a 10% running gel. Proteins were transferred to nitrocellulose membrane and the membrane was incubated successively at room temperature with 5% BSA in TTBS for 1 h. Membranes were incubated overnight at 4 °C with an anti-cPLA2, anti-COX-2, anti-phospho-p47^{phox}, anti-aquaporin-4 or anti-GAPDH according to the recommendation of manufacturer. Membranes were incubated with a 1:2000 dilution of anti-mouse or anti-rabbit horseradish peroxidase antibody for 1 h. The immunoreactive bands detected by ECL reagents were developed by Hyperfilm-ECL.

Total RNA extraction and gene expression analysis

Total RNA was extracted from A549 cells using Trizol, as previously described [5]. The cDNA containing 2 μg RNA was used as templates to analyze cPLA2 and COX-2 mRNA level. Oligonucleotide primers for β-actin, $cPLA_2$, and COX-2 were as follows: for β-actin: 5′-TGACGGGGTCACCCACACTGTGCCCATCTA-3′ (Sense), 5′-CTAGAAGCATTTGCGGTGGACGATG-3′ (Anti-sense); for cPLA2: 5′-CTCACACCACAGAA AGTTAAAAGAT-3′ (Sense), 5′-GCTACCACAGGCACATCA-CG-3′ (Anti-sense); for COX-2 : 5′-TTCAAATGAGATTGTGGGAAAATTGCT-3′ (Sense), 5′-AGATCATCTCTGCCTGAGTATCT-3′ (Anti-sense). The amplification profile included one cycle of initial denaturation at 94 °C for 5 min, 30 cycles of denaturation at 94 °C for 1 min, primer annealing at 58 °C (cPLA2), 62°C (COX-2), or 60 °C (β-actin), for 1 min, extension at 72 °C for 1 min, and then one cycle of final extension at 72 °C for 5 min. The expression of β-actin was used as an internal control for the assay of a constitutively expressed gene.

Cell number and cell viability determination

A549 cells were seeded in 6-cm dish or 24-well plates. The cells were serum-starved after growth confluence, and the PCS were added cells and incubated in CO2 incubator. At the end of incubation, 5 random pictures of cells were taken by HoloM4 monitor (Phase Holographic Imaging, Lung, Sweden), and the cell numbers were determined by holographic cell analysis. In addition, cell viability was analyzed by using XTT assay (Biological Industries., Connecticut, USA) according to the direction

of manufacturer. Absorbance was measured at 490 nm and 650 nm using a BioTek spectrophotometer (Bio Tek Instrument, Inc., Vmont USA).

ROS measurement by CM-H2DCFDA fluorescence

Formation of ROS in A549 cells was determined by the probe 2',7'-dichloro-fluorescin-diacetate (H_2DCF-DA), a non-fluorescent permeant molecule that passively diffuses into cells, fluorescence method. A549 cells (~90% confluence in 10-cm dishes) were loaded with 1 μM H_2DCF-DA for 30 min in PBS at 37 °C in a 95% air–5% CO_2 environment. The medium containing H_2DCF-DA was aspirated, and the cells were washed twice with PBS, and replenished with 5 mL of DMEM/F-12 medium. Cells were pretreated without or with the inhibitors for 1 h followed by exposure to PCS for the indicated time intervals. The cells were washed twice with ice-cold PBS and scraped using a lysis buffer (1X PBS containing 20% alcohol and 0.1% Tween 20). The cell lysates were transferred to 1.5-mL Eppendorf vials and centrifuged at 10,000 X g for 1 min at 4 °C. Fluorescence of oxidized DCF-DA, highly fluorescent 2',7'-dichlorofluorescein, in cell lysates, an index of formation of ROS, was measured by a VICTOR3 Multilabel Readers (PerkinElmer, Waltham, MA, U.S.) or Infinite® 200 PRO multimode reader (Tecan Group Ltd., Männedorf, Switzerland) with excitation and emission set at 490 and 530 nm, respectively.

Detection of mitochondrial activity

Activation of mitochondria increases the electron transfer of respiratory chain accompanied with increased ROS production. MitoSOX™ Red is a fluorogenic probe designed to measure ROS in mitochondria. MitoSOX™ Red reagent is live-cell permeant and is rapidly and selectively targeted to the mitochondria. Once in the mitochondria, MitoSOX™ Red reagent is oxidized by superoxide and exhibits red fluorescence. A549 cells were treated with PCS for the indicated time intervals, and MitoSOX™ Red Reagent was added at a final concentration of 5 μM to the cells and then incubated for 10 min at 37 °C. Subsequently, medium was removed, and the cells were washed thrice with PBS. The resulting fluorescence was measured using a VICTOR^3 Multilabel Readers with excitation and emission set at 510 and 580 nm, respectively.

Supplemental References

1 Chen, H. M. *et al.* AdipoR-increased intracellular ROS promotes cPLA2 and COX-2 expressions via activation of PKC and p300 in adiponectin-stimulated

human alveolar type II cells. *Am J Physiol Lung Cell Mol Physiol* **311**, L255-269, doi:10.1152/ajplung.00218.2015 (2016).

2 Huang, L., Scarpellini, A., Funck, M., Verderio, E. A. & Johnson, T. S. Development of a chronic kidney disease model in C57BL/6 mice with relevance to human pathology. *Nephron Extra* **3**, 12-29 (2013).

3 Neelisetty, S. *et al.* Renal fibrosis is not reduced by blocking transforming growth factor-beta signaling in matrix-producing interstitial cells. *Kidney Int* **88**, 503-514 (2015).

4 Zeniya, M. *et al.* The proteasome inhibitor bortezomib attenuates renal fibrosis in mice via the suppression of TGF-beta1. *Sci Rep* **7**, 13086 (2017).

5 Yang, C. M. *et al.* Cigarette smoke extract induces COX-2 expression via a PKCalpha/c-Src/EGFR, PDGFR/PI3K/Akt/NF-kappaB pathway and p300 in tracheal smooth muscle cells. *American journal of physiology. Lung cellular and molecular physiology* **297**, L892-902, doi:10.1152/ajplung.00151.2009 (2009).

Supplemental Table

Supplemental table S1. Comparisons of background bio-demographic characteristics and relevant laboratory data between selected uremic and non-uremic patients with pleural effusions.

	Uremic (n=20)		Non-Uremic (n=22)	
	n	(%)	n	(%)
**Patients**				
Sex (male/female)	12/8	(60.0)	10/12	(45.5)
Diabetes Mellitus	13	(65.0)	14	(63.6)
Hypertension	11	(55.0)	12	(54.5)
Coronary artery disease	7	(35.0)	8	(36.4)
Age	72.5 ± 13.3		82.0 ± 17.4	
SBP (mmHg)	137.7 ± 24.1		133.0 ± 21.5	
DBP (mmHg)	76.9 ± 13.2		75.9 ± 12.2	
WBC (per uL)	8029 ± 2355		7950 ± 3465	
BUN (mg/dl)	63.6 ± 28.7		20.6 ± 8.3	
Creatinine (mg/dl)	6.0 ± 2.0		0.9 ± 0.2	
eGFR (ml/min)	9.6 ± 5.8		72.5 ± 23.1	
**Pleural effusions**				
RBC (per uL)	6692.1 ± 13080.3		5940.4 ± 10912.5	
WBC (per uL)	613.1 ± 1372.8		628.3 ± 643.5	
Lymphocytes (%)	76.0 ± 24.1		79.1 ± 19.3	
Neutrophil (%)	24.0 ± 24.1		20.9 ± 19.3	
Protein (g/dL)	3.0 ± 1.5		1.9 ± 0.9	
PCS (ug/mL)	28.0 ± 29.2		5.1 ± 9.2	
IS (ug/mL)	27.1 ± 40.5		4.3 ± 8.8	
ENA78 (pg/ml)	1179.3 ± 2084.2		109.9 ± 150.4	
VEGF (pg/ml)	232.7 ± 184.3		132.1 ± 55.4	
IL8 (pg/ml)	1147.5 ± 107.1		917.6 ± 338.9	
MDC (pg/ml)	82.2 ± 114.3		16.7 ± 15.2	

Abbreviations: SBP, systolic blood pressure; DBP, diastolic blood pressure; Cr, Creatinine; eGFR, estimated glomerular filtration rate; BUN, blood urea nitrogen; WBC and RBC, white and red blood cells; PCS, p- cresyl sulphate; IS, indoxyl sulphate.

Note: the background bio-demographic characteristics were similar except the renal function related profiles between selected uremic and non-uremic patients.

Supplemental Figures and Figure Legends

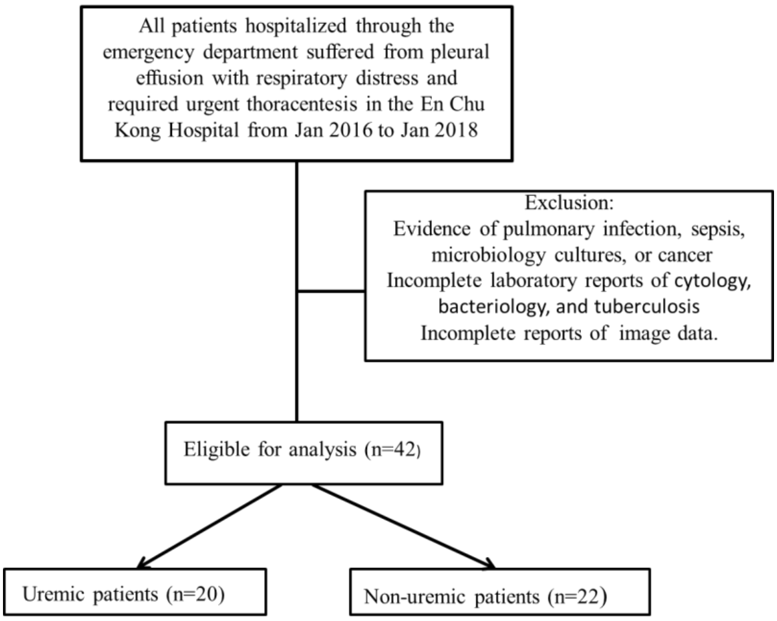

Supplemental Fig S1. The flow chart of patient enrollment. The selected patients with PE must receive complete surveys, including cytological and microbiological studies to exclude malignancy and pulmonary infection.

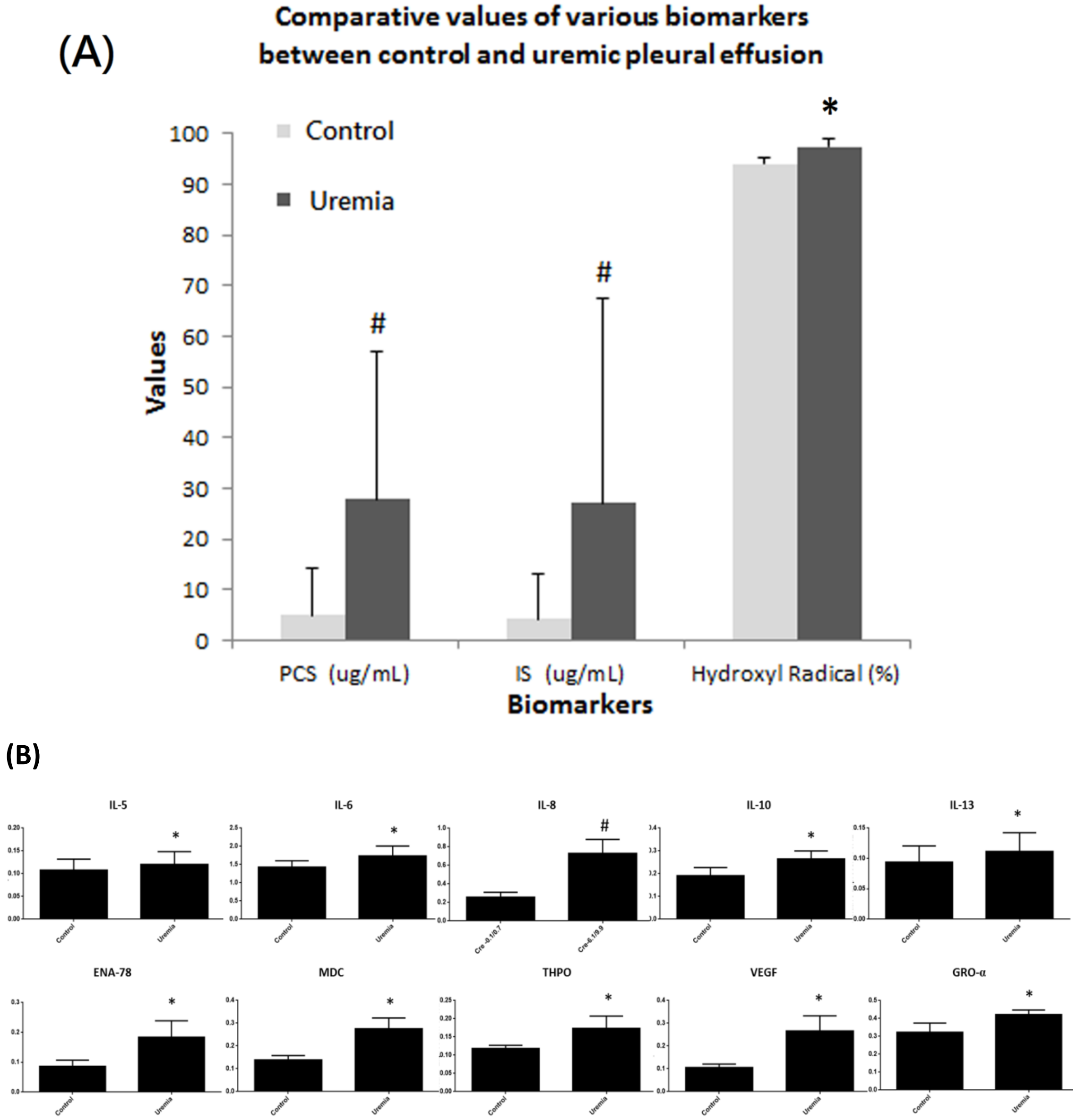

Supplemental Fig S2. (A) Pleural effusions from uremic patients exert higher concentrations of p-Cresyl sulfate (PCS), indoxyl sulphate (IS), and hydroxyl radicals than those from patients with pure cardiogenic pulmonary edema. The concentrations of PCS and IS were evaluated by Mass Spectrometer and hydroxyl radical concentrations were tested by thiobarbituric acid reaction.

(B) Enhanced expressions of multiplex human inflammatory cytokines in uremic pleural effusions. Relative expressions of human inflammatory cytokines were detected by RayBio® G-Series Human Cytokine Antibody Array 3 Kit. After screening 42 types of chemotactic cytokines, uremic pleural effusions enhanced expressions of IL-5, IL-6, IL-8, IL-10, IL-13, epithelial-derived neutrophil-activating peptide 78 (ENA-78), macrophage-derived chemokine (MDC), thrombopoietin, vascular

endothelial growth factor (VEGF), and growth-related oncogene-α (GRO-α). Data are expressed as mean ± SEM; n=42; **, P<0.05; and #, P<0.01;* respectively.

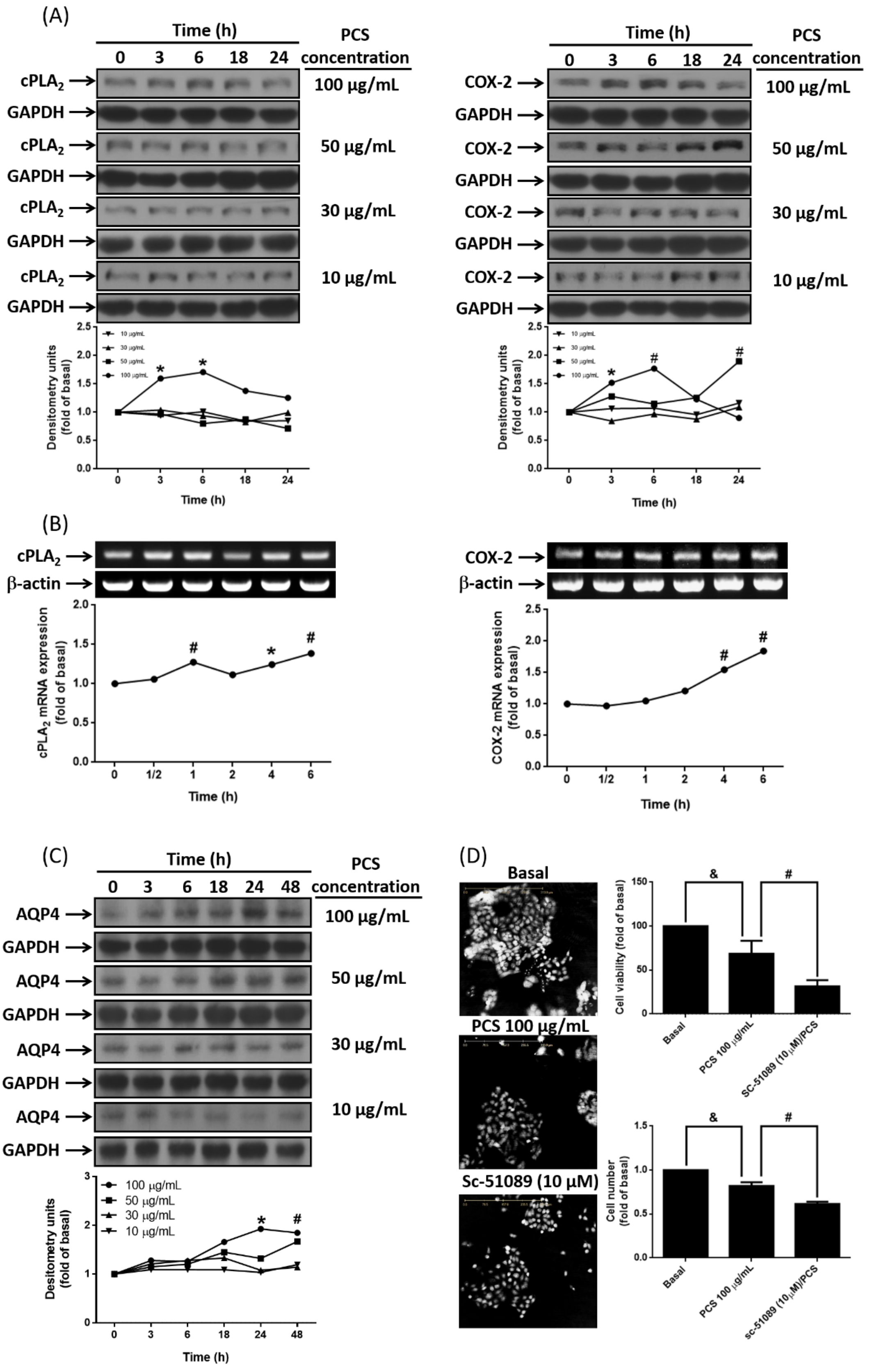

Supplemental Fig S3. PCS enhances protein and mRNA expression of cPLA2, COX-2 and aquaporin-4 in human pulmonary alveolar cells. Cells were stimulated by

various concentrations of PCS for the different time intervals. Effects of PCS on cPLA2 and COX-2 (A) protein expression were determined by Western blot and (B) mRNA expressions were detected by RT-PCR. (C) aquaporin-4 expression was detected by Western blot with anti-aquaporin-4 antibody. (D) A549 cells were pretreated without or with 10 μM sc-51089 (inhibitor of PGE2 receptor), and then incubated with 100 μg/mL PCS for 72 h. Cell viability and cell number were analyzed by XTT assay and Holographic cell analysis. Data are expressed as line chart or mean ± SEM of different independent experiments (n>4); *& P<0.05, *P<0.05, #P<0.01* as compared with the group of 0 h or between the two indicated groups.
